# High-Throughput Fabrication of Antibacterial Starch/PBAT/AgNPs@SiO_2_ Films for Food Packaging

**DOI:** 10.3390/nano11113062

**Published:** 2021-11-14

**Authors:** Shengxue Zhou, Xiaosong Zhai, Rui Zhang, Wentao Wang, Loong-Tak Lim, Hanxue Hou

**Affiliations:** 1 College of Food Science and Engineering, Shandong Agricultural University, Engineering and Technology Center for Grain Processing of Shandong Province, Tai’an 271018, China; zhoushengxue666@163.com (S.Z.); xszhai@126.com (X.Z.); xuyuyisha@163.com (R.Z.); wangwt@sdau.edu.cn (W.W.); 2 Department of Food Science, University of Guelph, Guelph, ON N1G 2W1, Canada

**Keywords:** starch, PBAT, antibacterial films, high-throughput fabrication, food packaging

## Abstract

In this current work, antimicrobial films based on starch, poly(butylene adipate-*co*-terephthalate) (PBAT), and a commercially available AgNPs@SiO_2_ antibacterial composite particle product were produced by using a melt blending and blowing technique. The effects of AgNPs@SiO_2_ at various loadings (0, 1, 2, 3, and 4 wt%) on the physicochemical properties and antibacterial activities of starch/PBAT composite films were investigated. AgNPs@SiO_2_ particles were more compatible with starch than PBAT, resulting in preferential distribution of AgNPs@SiO_2_ in the starch phase. Infusion of starch/PBAT composite films with AgNPs@SiO_2_ marginally improved mechanical and water vapor barrier properties, while surface hydrophobicity increased as compared with films without AgNPs@SiO_2_. The composite films displayed superior antibacterial activities against both Gram-positive (*Staphylococcus aureus*) and Gram-negative (*Escherichia coli*) bacteria. The sample loaded with 1 wt% AgNPs@SiO_2_ (SPA-1) showed nearly 90% inhibition efficiency on the tested microorganisms. Furthermore, a preliminary study on peach and nectarine at 53% RH and 24 °C revealed that SPA-1 film inhibited microbial spoilage and extended the product shelf life as compared with SPA-0 and commercial LDPE packaging materials. The high-throughput production method and strong antibacterial activities of the starch/PBAT/AgNPs@SiO_2_ composite films make them promising as antimicrobial packaging materials for commercial application.

## 1. Introduction

Packaging plays an important role in controlling food spoilage during distribution and extending product shelf life [1]. Advances in materials science and consumer concerns on food additives have spurred the development of novel active packaging materials. In particular, nanotechnology provides a useful platform for the development of novel antibacterial materials by changing their physical and chemical characteristics [2,3].

Metals or metal oxides are antimicrobial agents that are promising for antibacterial packaging applications. For example, silver is a strong biocide that acts strongly against a broad spectrum of bacteria and fungi. Silver containers were used to delay the spoilage of food more than 1000 years ago, before the advent of modern preservation technologies. The antibacterial effect of silver can be attributed to the gradual release of Ag ions [4,5]. Moreover, metallic silver can display enhanced antibacterial activity when reduced to the nano-scale [6]. Compared with metallic silver, silver nanoparticles (AgNPs) exhibit antibacterial activity at low concentrations due to their large active surface area and high surface charge density [7]. These properties are desirable for antibacterial applications wherein low loadings are needed to achieve antimicrobial efficacy while avoiding undesirable side effects (e.g., toxicity, off-flavor) [8,9].

At present, various silver-based commercial products are available (e.g., Aquacel Ag^®^, Kaltostat^®^, Bactigras^®^, Quench^®^) and have been approved by the United States Food and Drug Administration (FDA) and the European Food Safety Authority (EFSA) for antibacterial applications [10,11]. These AgNP products are designed for the production of antibacterial composites with enhanced material properties, by allowing optimal dispersion in carrier substrates at a low concentration, and are able to withstand high-temperature processing conditions [12,13]. These additives can result in a reduction of permeability of gases and moisture due to increased tortuosity of diffusion paths of the penetrating molecules, thereby enhancing the barrier properties of the composite as compared with the neat polymers. In addition, the interaction between the polymer chains and the filler particles reduces the segmental movements and contributes to enhanced mechanical strength and thermal resistance of the composite [14,15].

Commercially available petrochemical-based packaging materials infused with AgNPs prepared by extrusion blowing, such as poly(vinyl chloride) [16,17], polyethylene [18,19], and polystyrene [20], have been investigated by various researchers. However, these polymers are non-biodegradable. Moreover, their relatively high moisture barrier properties tend to result in high headspace relative humidity in packages for fresh produce, which may lead to a rapid proliferation of spoilage microorganisms [21]. Hence, researchers have developed packaging materials derived from biodegradable polymers incorporated with AgNPs for antibacterial applications [22,23]. However, most of these studies involved processing techniques that are not conducive for high throughput industrial production (e.g., solution casting, hot pressing, or others) [24,25].

We previously reported a study on the production of extrusion-blown biodegradable poly(butylene adipate-*co*-terephthalate) (PBAT)/starch blend films with material properties that were promising for food packaging [26]. Barrier properties against H_2_O, CO_2_ and O_2_ of these films could be adjusted by varying the blend ratio of the two polymers for specific applications. For example, the polymer blend ratio can be optimized to reduce the respiration rate of fresh produce while preventing anaerobic respiration and delaying senescence. These characteristics are desirable for packaging fresh fruits with strong respiration that are susceptible to microbial spoilage [27]. The application of antimicrobial AgNPs to PBAT/starch composite films, produced using industrial-representative extrusion-blown processes, could further enhance the preservation of these highly perishable products.

The primary objective of this work was to develop starch/PBAT/AgNPs@SiO_2_ composite films by using extrusion melt blending and blowing techniques. The effects of AgNPs@SiO_2_ loadings (0, 1, 2, 3, and 4 wt%) on the physicochemical properties and antibacterial activities of the composite films were investigated. Preliminary packaging studies on peach and nectarine were conducted using the composite films under simulated storage conditions to evaluate their potential for commercial food packaging applications.

## 2. Materials and Methods

### 2.1. Materials

**Figure 1:**
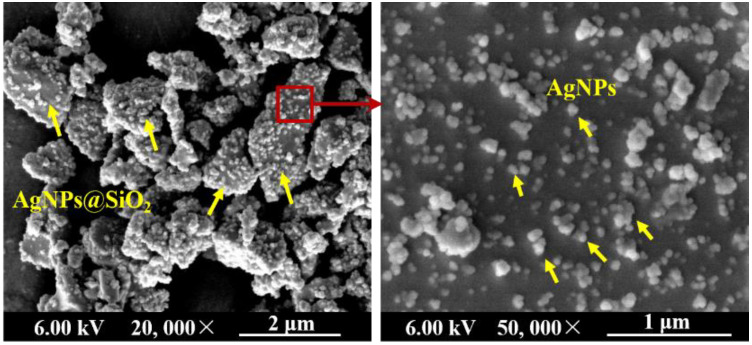
Micromorphology of the commercial AgNPs@SiO_2_ antibacterial particles under the magnification of 20,000× and 50,000×.

Modified cassava starch (hydroxypropyl distarch phosphate, mode HP-CF T0278) was purchased from Puluoxing Starch Co., Ltd. (Hangzhou, China), with the hydroxypropyl group content of 3.1% and moisture content of 12.5%. PBAT (Biocosafe™2003 F) was manufactured by Xinfu Pharmaceutical Co., Ltd. (Hangzhou, China) with a density of 1.25 g/cm^3^ and a melting point of 115 °C. Glycerol (analytical grade) was obtained from Kaitong Chemical Reagent Co., Ltd. (Tianjin, China). A commercial antimicrobial nanocomposite product (JW-02-JK-1060; Figure 1), comprising silicon dioxide particles loaded with 10 wt% AgNPs (AgNPs@SiO_2_), was purchased from Jinwei Nano New Material Co., Ltd. (Hangzhou, China). Tryptone was acquired from Aoboxing Bio-Tech Co., Ltd. (Beijing, China). Agar powder was produced by Solarbio Science & Technology Co., Ltd. (Beijing, China). Yeast extract was yielded by Oxoid. Co., Ltd. (Basingstoke, UK). All other reagents were of analytical grade and commercially available. *E. coli* (CVCC1387) was provided by the Agricultural Culture Collection of China, and *S. aureus* (CMCC26003) was maintained in the College of Food Science and Engineering, Shandong Agricultural University.

### 2.2. Preparation of Starch/PBAT/AgNPs@SiO_2_ (SPA) Films

Film preparation was based on the method reported in our previous work [26], with some modifications. HPDSP (400 g), PBAT (1600 g), glycerol (140 g), and AgNPs@SiO_2_ were mixed together in a mixer (SHR-50A, Hongji Machinery, Zhangjiagang, China) at room temperature for 10 min. The resulting mixtures were extruded using a twin-screw extruder (SHJ-20B; Giant Machinery, Nanjing, China) with a screw diameter (D) of 21.7 mm and length of 40D. The temperature profile of the extruder, from the feeding throat to the die, was 110, 120, 130, 135, 125, and 110 °C. The screw speed was set at 170 rpm. The extruded strand was air-cooled and then cut into pellets. Films were prepared using a laboratory film-blowing extruder (SCM-50, Lianjiang Machinery, Zhangjiagang, China) with a with a screw diameter (D) of 25 mm, length of 30D, and annular die diameter of 30 mm. The temperature profile of the extruder, from the feeding zone to the die, was 120, 130, 135, 145, and 135 °C. The screw speed was set at 30 rpm. Film samples loaded with 0, 1, 2, 3, and 4 wt% AgNPs@SiO_2_ (based on the total weight of starch and PBAT), were coded as SPA-0, SPA-1, SPA-2, SPA-3, and SPA-4, respectively.

### 2.3. Characterization of Films

#### 2.3.1. Scanning Electron Microscopy (SEM)

Cross-sectional and surface morphology of films were investigated using a scanning electron microscope (Quanta FEG 250, FEI, OR, USA) operating at an acceleration voltage of 6.0 kV. The cross-sectional samples were obtained by freezing the films with liquid nitrogen and then fracturing them. Samples were fixed on the carbon tape and examined after sputter-coating with gold.

#### 2.3.2. Attenuated Total Reflectance-Fourier Transform Infrared (ATR-FTIR) Spectroscopy

ATR-FTIR spectra of films were measured using an FTIR spectrometer (Nicolet iS5, Thermo Fisher Scientific, MA, USA) equipped with an ATR sampling accessory. The sample was placed on the ATR crystal and measured in the wavenumber range of 4000–550 cm^−1^. The cumulative numbers of scan and scanning resolution were 30 and 4 cm^−1^, respectively.

#### 2.3.3. Differential Scanning Calorimetry (DSC)

Thermal properties of films were determined by a DSC (200PC, NETZSCH, Selb, Germany). Samples were sliced into round pieces, and 10–15 mg samples were placed in aluminum pans. The pan was sealed and then loaded onto the stage. An empty pan was used as a reference. The samples were scanned from 25 to 150 °C at a rate of 10 °C/min under a nitrogen atmosphere.

#### 2.3.4. Oxygen Permeability (OP)

OP values of films were obtained by a film permeability tester (BTY-B1, Labthink Instruments, Jinan, China) at 25 °C and 53% RH. Each film was cut into a circle with a diameter of 100 mm with a test area of 38 cm^2^. Based on constant-volume-variable-pressure method, OP values were determined by dividing the oxygen transmission rate by the partial oxygen pressure difference between the two sides of the film and multiplying this number by the average thickness of the film. OP values were obtained from triplicate measurements.

#### 2.3.5. Water Vapor Permeability (WVP)

WVP values of films were measured by an automatic WVP tester (PERME™ W3/030, Labthink Instruments, Jinan, China) on the basis of ASTM E96/E96M-16 [27]. The film was cut (80 mm in diameter) and then put into the measuring cups. The tests were performed at 38 °C and 90% RH after 4 h of equilibration, and a weighing interval of 2 h was conducted during 12 h of testing time. WVP values of each sample were determined from three measurements.

#### 2.3.6. Water Contact Angle (WCA)

A contact-angle goniometer (JC-2000C1, Zhongchen, Shanghai, China) was used to measure the WCA of films to evaluate their surface hydrophobicity. The dynamic sessile drop method according to the triple solid-liquid-gas interface principle was used for observing and recording the WCA on the films. Approximately 7 μL of deionized water was dropped onto the sample surface using a precision microsyringe, and at the same time, a photo was immediately taken by a high-speed charge coupled device camera. WCA was analyzed using JC2000 software.

#### 2.3.7. Mechanical Properties

Tensile strength (TS, MPa) and elongation at break (EAB, %) of films were performed according to ASTM D882-12 [28,29] using a tensile tester (PARAM™ XLW, Labthink Instruments, Jinan, China). All films were cut into strips (120 mm × 15 mm) and equilibrated at 23 °C and 53% RH for 72 h before testing [30]. The initial distance between the grips was 50 mm, and the extensional speed was set at 100 mm/min. Each test was repeated six times.

#### 2.3.8. Antimicrobial Activity

The antibacterial activity of SPA films was evaluated by a plate counting method [31]. *S. aureus* and *E. coli* were inoculated in liquid medium (Luria-Bertani) separately and cultivated in a shaker at 37 °C for 24 h. The obtained culture broth was diluted with 0.9% NaCl. The antimicrobial films were placed into a 10 mL centrifuge tube, and then 50 μL of bacterial solution (10^6^ CFU/mL) was dropped. Subsequently, the above was incubated in an incubator at 37 °C for 3 h. The bacteria were washed with PBS buffer, applied evenly on solid medium (Luria-Bertani with nutrient agar), and then incubated at 37 °C for 24 h. The number of colonies was then enumerated. *Antibacterial efficiency* (%) was calculated by the following equation:

$$Antibacterial\;efficiency\;\left(\%\right)=\frac{x-y}{x}$$

where *x* is number of colonies on SPA-0 and *y* is the number of colonies on antibacterial films.

### 2.4. Preliminary Packaging Studies on Peach and Nectarine

Nectarines (*Prunus persica var. nectarina*) and peaches (*Amygdalus persica* L. *Batsch*) were purchased from a local supermarket in Tai’an, China. Coherent films with uniform thickness (90–100 μm) were trimmed into 23 cm × 24 cm and then sealed on three sides using a packaging machine (Xingduo, Shanghai, China) to form prefabricated bags. Peaches and nectarines of similar maturity and size without visible pest and mechanical damage were packaged separately (i.e., one fruit per bag) in the prefabricated bags and stored at 53% RH and 24 °C. The treatments tested were (i) unpackaged fruits, (ii) low-density polyethylene (LDPE) bag, (iii) SPA-0 bag, and (iv) SPA-1 bag.

### 2.5. Statistical Analysis

The statistical significance of each value was analyzed using ANOVA (SPSS 21, IBM, NY, USA). The data were expressed as the mean ± standard deviation (SD). Comparisons among the mean values were determined using Tukey’s multiple range tests at a 5% significance level.

## 3. Results and Discussion

### 3.1. Morphology

**Figure 2:**
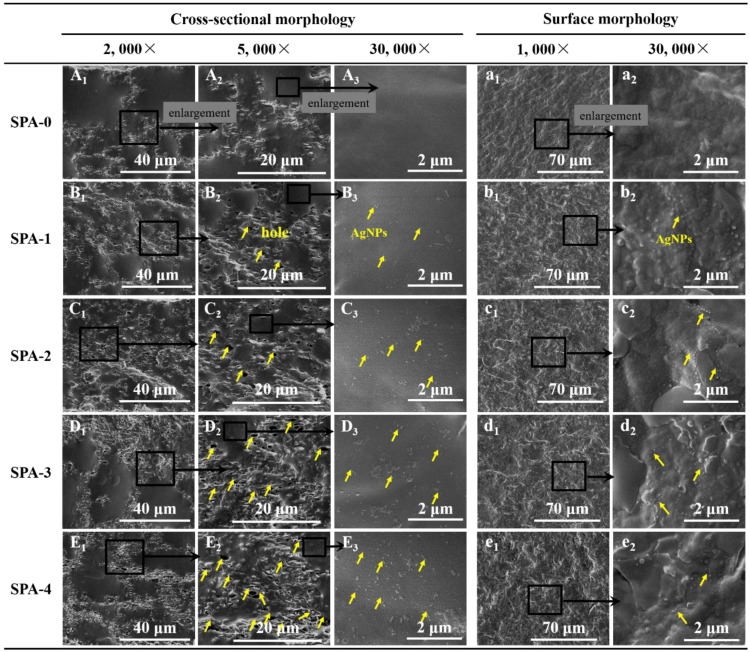
SEM images of starch/PBAT antibacterial films loaded with different levels of AgNPs@SiO_2_.

Cross-sectional morphologies of SPA films are shown in Figure 2. The PBAT appeared as the continuous phase (dark region) while the dispersed starch-rich phase appeared as bright regions (Figure 2(A_1_–E_1_)). This observation shows that the two phases are thermodynamically incompatible. Holes were noticeable in the starch phase at higher magnification (Figure 2(A_2_–E_2_)), but were largely absent in the PBAT phase. These holes were presumably caused by the partial shedding of AgNPs@SiO_2_ particles during cryo-fracturing. The preferential partition of AgNPs@SiO_2_ in the starch phase might be due to their similar polarity and compatibility. Furthermore, as the AgNPs@SiO_2_ loading increased, an increase in the number of holes with enlarged sizes was observed (Figure 2(A_2_–E_2_)) due to self-agglomeration.

The extensive distribution and loose binding of AgNPs@SiO_2_ in the starch phase are important to promote their rapid diffusion and migration along with starch from the blend matrix in the antibacterial measurement scenarios, thereby achieving antibacterial effects. At the highest magnification (Figure 2(A_3_–E_3_)), the distribution of AgNPs@SiO_2_ in the PBAT continuous phase showed a similar distribution trend. The incorporation of AgNPs@SiO_2_ also resulted in the continuity and compactness of the film matrix, which further affected the mechanical and barrier properties. In terms of surface morphology, SPA-0 film had the smoothest surface topography as shown in Figure 2a, but film surfaces became rougher (Figure 2b–e) as AgNPs@SiO_2_ loading increased, due to an increased agglomeration of the AgNPs@SiO_2_ particles.

### 3.2. ATR-FTIR

**Figure 3:**
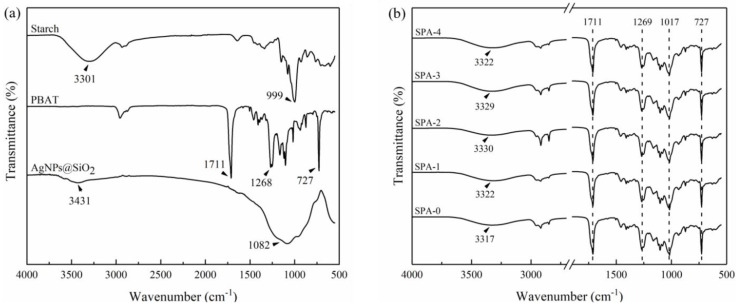
ATR-FTIR spectra of (**a**) starch, PBAT, and AgNPs@SiO_2_; and (**b**) starch/PBAT antibacterial films loaded with different levels of AgNPs@SiO_2_.

ATR-FTIR spectra of starch, PBAT, and AgNPs@SiO_2_ are shown in Figure 3a. The broad band at 1082 cm^−1^ corresponds to the Si-O-Si anti-symmetric stretching vibration of the silicon-oxygen tetrahedron in the crystalline silica, while the symmetric stretching vibration band at 999 cm^−1^ is due to the C-O-C stretching in starch glycosidic bonds [14,32]. The two bands overlapped at around 1017 cm^−1^ in the blend film. The band at 3431 cm^−1^ for AgNPs@SiO_2_ is attributed to the stretching vibration of the bound water in the AgNPs@SiO_2_ particles [33]. This band appeared next to the stretching vibration at 3301 cm^−1^ related to O-H in starch; the band here coincided around 3320 cm^−1^ after film formation [34]. The similar polarity between starch and AgNPs@SiO_2_ particles might have resulted in their preferential dispersion in the starch phase. As shown in Figure 3b, the band shifted to higher wavenumbers as AgNPs@SiO_2_ loading in the films increased. According to the harmonic-oscillator model, the peak wavenumber correlates negatively with the molecular interaction [35]. The peak of the hydroxyl group shifted to a higher wavenumber as the AgNPs@SiO_2_ loading increased for SPA-1 and SPA-2 films, indicating that AgNPs@SiO_2_ disrupted the interaction between the hydroxyl groups of starch molecules. As the loading of AgNPs@SiO_2_ increased to 3 wt%, the shift of this peak to a lower wavenumber could be attributed to the self-aggregation of the particles, as reported in the literature [31,36]. The peaks at 1711 and 1268 cm^−1^ in Figure 3a are attributed to stretching vibrations of C=O and C-O bonds in the ester group of PBAT, respectively, and the peak at 727 cm^−1^ is related to stretching vibrations of multiple adjacent methyl groups from the polymer backbone chain [24,37]. It can be seen from Figure 3b that the introduction of AgNPs@SiO_2_ resulted in negligible changes to the position and intensity of the characteristic peaks of PBAT, indicating minimal interaction of the particles with PBAT [31].

### 3.3. DSC

**Figure 4:**
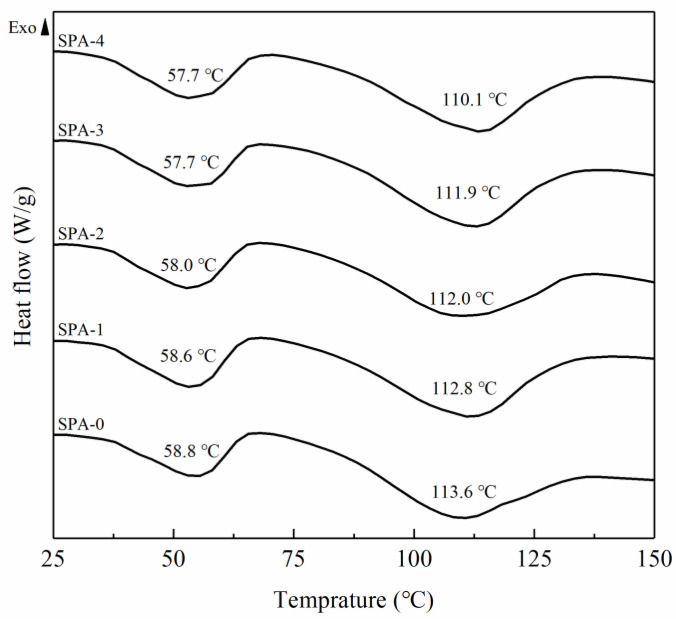
DSC thermograms of starch/PBAT antibacterial films loaded with different levels of AgNPs@SiO_2_.

DSC thermograms of SPA films loaded with different levels of AgNPs@SiO_2_ are illustrated in Figure 4. PBAT is a block co-polyester composed by repetition units of aliphatic butylene adipate (BA) and aromatic butylene terephthalate (BT) [31]. The incompatibility between BA and BT segments tends to cause microphase separation and agglomeration into soft and hard domains in the polymer, therefore resulting in two endothermic peaks in the thermograms at around 60 and 115 °C, respectively [34,38,39]. As shown in Figure 4, the melting temperatures (T_m_) corresponding to the BA and BT segments in the control (SPA-0) appear at 58.8 and 113.6 °C, respectively. The T_m_ associated with the flexible BA segment decreased to 57.7 °C as AgNPs@SiO_2_ loading increased, while the T_m_ corresponding to the rigid BT segment increased to 110.1 °C. The variation of endothermic peaks in DSC thermograms depends on the order of the polymer chain; the higher the T_m_, the more ordered the crystalline structures [32]. The insertion of AgNPs@SiO_2_ into the film matrix might have disrupted the recrystallization process, thereby leading to the decreased T_m_ [12].

### 3.4. Mechanical Properties

Mechanical properties of SPA films are summarized in Table 1. As shown, TS and EAB values increased and then decreased with increasing AgNPs@SiO_2_ loading. The micro-sized AgNPs@SiO_2_ particles, due to their high specific surface area, could interact strongly with polymer chains to improve the mechanical properties of SPA films [28]. These particles effectively transferred the stress by absorbing deformation work [25,36,40]. However, the material enhancement effect also depended on how uniformly the particles were dispersed in the film matrix [28]. These decreasing TS and EAB trends for composite SPA films at elevated AgNPs@SiO_2_ loadings were caused by the self-aggregation of these particles above 2 wt% AgNPs@SiO_2_ loading levels. The aggregation of the particles could reduce their exposed surface area and available binding sites with the polymer chains, thereby reducing the interaction between the two phases and disrupting the structural continuity of the film matrix [31,41].

### 3.5. Barrier Properties

Water vapor and oxygen barrier properties are important in food packaging applications. As shown in Table 1, WVP and OP values decreased and then increased with increasing AgNPs@SiO_2_ loading. The minimum WVP and OP values were observed at 2 wt% loading, indicating that the SPA-2 film had the strongest barrier properties among the films tested. AgNPs@SiO_2_ particles that distributed in the film matrix increased the diffusion path tortuosity of water and oxygen molecules through films [42]. Peighambardoust et al. [28] reported that the electrostatic interaction between the positively charged AgNPs and hydroxyl groups reduces the number of free hydroxyl groups, thereby reducing the penetration of water molecules. As AgNPs@SiO_2_ loading further increased from 2 to 4 wt%, the WVP and OP values increased, indicating a decrease in barrier properties of the films, which could be attributed to the self-aggregation of AgNPs@SiO_2_ particles [43,44]. This result is in accordance with the SEM micrographs (Figure 2C–E). Yoksan et al. [45] proposed that high loading of AgNPs@SiO_2_ particles could hinder the formation of intermolecular hydrogen bonds between the substrates, leading to incompatibility of the substrates and thereby weakening their barrier properties.

### 3.6. Surface Hydrophilicity

**Figure 5:**
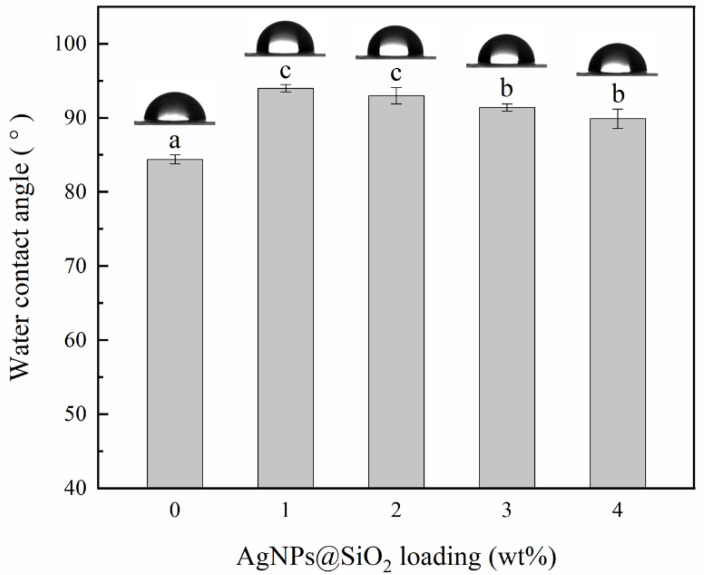
Water contact angle of starch/PBAT antibacterial films loaded with different levels of AgNPs@SiO_2_. ^a–c^: Different letters within the same column indicate significant differences among the samples (*p* < 0.05).

WCA measures the surface hydrophilicity of packaging materials, which reflects their wettability [46]. As shown in Figure 5, the incorporation of AgNPs@SiO_2_ particles increased the WCA of films at all loading levels. As mentioned earlier, the interaction between AgNPs with the hydroxyl groups could reduce the free hydroxyl groups, thereby reducing the penetration of water molecules [44,47]. Strong interactions between AgNPs@SiO_2_ particles and polymer chains in SPA films at low loading promoted the formation of a dense network. Furthermore, the SPA film surface also became rougher on account of the incorporation of AgNPs@SiO_2_ particles, which was beneficial to increasing WCA, as described by Panrong et al. [48]. However, the self-aggregation behavior caused by too many AgNPs@SiO_2_ particles had a negative impact, which led to a decrease in WCA.

### 3.7. Antimicrobial Activity

**Figure 6:**
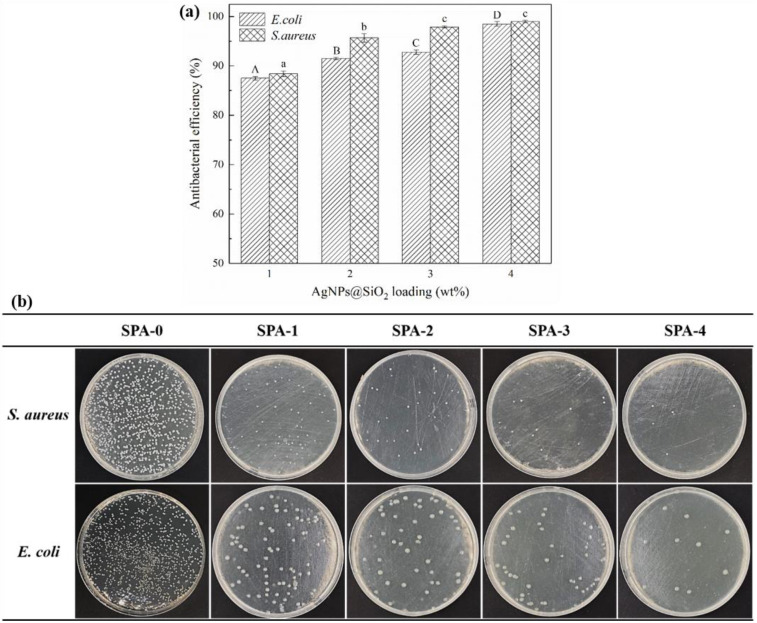
Antibacterial efficiency (**a**) and representative photos (**b**) of antibacterial testing of antibacterial films against *S. aureus* and *E. coli* after 3 h of contact. Results are reported as means ± standard deviation of triplicate determinations. A–D and a–c: Different designations within the same tested microorganism indicate significant differences among the samples (*p* < 0.05).

The antibacterial efficiency of SPA films loaded with different levels of AgNPs@SiO_2_ particles against the tested microorganisms is presented in Figure 6. As shown, substantial decreases in bacterial colonies were observed on the samples loaded with AgNPs@SiO_2_ as compared with the control (SPA-0), and the antibacterial efficiency was positively correlated with the loading. The antibacterial films loaded with AgNPs inhibit microorganisms in the surrounding environment mainly through the migration of silver, which is likely to be made up of both nanoparticles and ions. AgNPs are able to alter the permeability of cell membranes, leading to the loss of many nutrients and metabolite losses [4]. More importantly, AgNPs can also enter the cell via the “Trojan horse effect” and afterwards are oxidized to release Ag^+^, which enables the presence of both AgNPs and Ag^+^ in cells [5,49]. AgNPs can directly bind to RNA polymerase and inhibit gene transcription through the “particle effect”. The presence of Ag^+^ can also increase the reactive oxygen species and induce oxidative stress responses, which damage the cells and eventually cause death [3,31]. In this present study, SPA-1 film achieved nearly 90% inhibition efficiency on the tested microorganisms within 3 h, which was higher than other antibacterial films loaded with the same level of AgNPs [16]. The reason might be that AgNPs@SiO_2_ particles tend to disperse in the starch phase but weakly bind to the starch matrix, which promotes the rapid diffusion and desorption of silver from the matrix along with starch.

Studies on dispersing AgNPs into synthetic polymers (e.g., PE, PLA, PBAT) to impart antibacterial activity have been extensively reported [4,24,25,46,50]. As mentioned by Zhang et al. [31], the film obtained by combining PBAT with AgNPs reached an antibacterial rate of more than 90% after 24 h of exposure to the test *S. aureus* and *E. coli*. In the present study, the film loaded with the same mass ratio of AgNPs reached the same antibacterial rate after being in contact with microorganisms for 3 h. This might be caused by the incorporation of starch into the PBAT film, which facilitated the rapid release of silver.

### 3.8. Preliminary Packaging Studies on Peach and Nectarine

**Figure 7:**
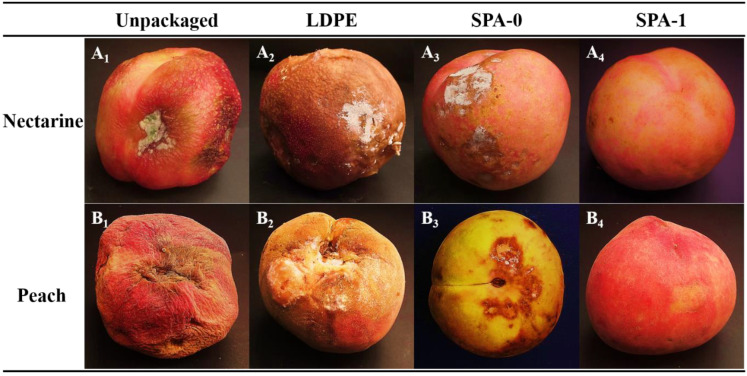
Photographs of nectarines and peaches after storage at 53% RH and 24 °C. (**A_1–4_**): Photographs of nectarines after 9 days of storage in different types of packaging bags. (**B_1–4_**): Photographs of peaches after 14 days of storage in different types of packaging bags.

To explore the potential application of the SPA films to the antibacterial packaging of fresh produce, nectarines and peaches were stored in sealed bags made of the SPA-0, SPA-1, or LDPE films. Unpackaged fruits were used as a control. Figure 7 shows photographs of nectarines and peaches stored in different packaging for 9 and 14 d for visual comparison. The unpackaged samples showed obvious wilting and and fungal growth (Figure 7(A_1_,B_1_)). The LDPE packaged samples showed substantial decays (Figure 7(A_2_,B_2_)), whereas the samples stored in the SPA-0 film had lower extents of decay (Figure 7(A_3_,B_3_)). The higher WVP of starch/PBAT composite films than the LDPE film might have resulted in lower headspace relative humidity that delayed the growth of microorganisms. However, none of the samples stored in the SPA-1 film showed signs of microbial growth or wilting (Figure 7(A_4_,B_4_)), although slight browning was detected on the nectarine skin but not on the peaches. This was mainly due to the reaction intensity of various fruits in producing O_2_ and CO_2_ during storage and the different degrees of tolerance to gas composition [49,51,52,53]. These results suggest that the SPA films have great potential in inhibiting the spoilage of fresh fruits and extending their shelf life.

## 4. Conclusions

A high-throughput process was developed for the production of starch/PBAT composite films infused with commercially available AgNPs@SiO_2_ particles via melt blending and blowing techniques. SEM micrographs revealed that at a 1 wt% level, AgNPs@SiO_2_ particles were evenly distributed in the matrix, and the antibacterial films loaded with 2–4 wt% AgNPs@SiO_2_ exhibited different degrees of aggregation behavior. At 1 to 2 wt% loading levels, the AgNPs@SiO_2_ improved the general material properties of starch/PBAT films, but the self-aggregation at higher loading levels did not result in further enhancement of material properties. Antibacterial experiments demonstrated that the film loaded with 1 wt% AgNPs@SiO_2_ produced nearly 90% inhibition efficiency against both *S. aureus* and *E. coli* within 3 h. Future research is needed to elucidate the migration and diffusion of silver as affected by the starch content. Preliminary packaging studies on peach and nectarine revealed that the SPA-1 film exhibited stronger preservation effects than the SPA-0 and commercial LDPE films. These results suggest that dispersing AgNPs@SiO_2_ particles in starch/PBAT films is a promising approach to develop antibacterial films for commercial food packaging.

## Figures and Tables

**Table 1:** Mechanical and barrier properties of starch/PBAT antibacterial films loaded with different levels of AgNPs@SiO_2_.

Samples	Mechanical Properties		Barrier Properties	
	TS (MPa)	EAB (%)	WVP (×10^−11^ g·m·m^−2^·s^−1^·Pa^−1^)	OP (×10^−14^ cm^3^·cm·cm^−2^·s^−1^·Pa^−1^)
SPA-0	10.27 ± 0.55 ^a,b^	656.63 ± 11.98 ^a^	3.58 ± 0.07 ^b^	6.26 ± 0.02 ^b^
SPA-1	10.91 ± 0.66 ^b^	646.53 ± 7.39 ^a^	3.09 ± 0.08 ^a^	5.37 ± 0.20 ^a^
SPA-2	11.29 ± 0.45 ^b^	774.40 ± 76.69 ^b^	3.07 ± 0.12 ^a^	5.48 ± 0.32 ^a^
SPA-3	10.30 ± 0.45 ^a,b^	760.00 ± 37.76 ^b^	3.09 ± 0.17 ^a^	5.48 ± 0.53 ^a^
SPA-4	9.64 ± 0.68 ^a^	751.00 ± 21.96 ^b^	3.14 ± 0.04 ^a^	6.31 ± 0.08 ^b^

Results are quoted as the mean ± standard deviation of six replicates. ^a,b^: Different letters within the same column indicate significant differences among the samples (*p* < 0.05).

## Data Availability

The data presented in this study are available on request from the corresponding author.
